# Safety and efficacy of daratumumab in Chinese patients with relapsed or refractory multiple myeloma: a phase 1, dose-escalation study (MMY1003)

**DOI:** 10.1007/s00277-022-04951-3

**Published:** 2022-10-27

**Authors:** Hongmei Jing, Li Yang, Junyuan Qi, Lugui Qiu, Chengcheng Fu, Junmin Li, Min Yang, Ming Qi, Ni Fan, Jia Ji, Jiajia Lu, Yunan Li, Jie Jin

**Affiliations:** 1 Peking University Third Hospital, Beijing, China; 2 State Key Laboratory of Experimental Hematology, National Clinical Research Center for Hematological Disorders, Institute of Hematology and Blood Diseases Hospital, Chinese Academy of Medical Sciences and Peking Union Medical College, Tianjin, China; 3 The First Affiliated Hospital of Soochow University, Suzhou, China; 4 Rui Jin Hospital, Shanghai Jiao Tong University School of Medicine, Shanghai, China; 5 The First Affiliated Hospital of Zhejiang University College of Medicine, 79 Qingchun Rd, Hangzhou, 310003 China; 6 Janssen Research & Development, LLC, Spring House, PA USA; 7 Janssen Research & Development, LLC, Shanghai, China; 8 Janssen Research & Development, LLC, Beijing, China

**Keywords:** Monoclonal antibody, CD38, Hematologic malignancies, Plasma cell disorders, Monotherapy

## Abstract

Daratumumab monotherapy demonstrated favorable safety and efficacy in relapsed/refractory multiple myeloma (RRMM) patients in the global phase 1/2 GEN501 and phase 2 SIRIUS studies. MMY1003 evaluated daratumumab monotherapy specifically in Chinese patients with RRMM. This 3-part, open-label, phase 1, dose-escalation study included patients with ≥ 2 prior lines of therapy. Part 3 included patients who had received a proteasome inhibitor (PI) and immunomodulatory drug (IMiD) and experienced disease progression on their last regimen. Patients received intravenous daratumumab 8 mg/kg or 16 mg/kg in part 1 and 16 mg/kg in parts 2 + 3. Primary endpoints were dose-limiting toxicity (DLT; part 1), pharmacokinetics (parts 1 + 2), and adverse events (AEs). Fifty patients enrolled. The first 3 patients in part 1 received daratumumab 8 mg/kg; remaining patients in parts 1–3 received daratumumab 16 mg/kg. In the daratumumab 16 mg/kg group (*n* = 47), patients received a median of 4 prior lines of therapy; 32% were refractory to a PI and IMiD, and 79% were refractory to their last prior therapy. No DLTs occurred. Thirty-six (77%) patients reported grade 3/4 treatment-emergent AEs. Thirteen (28%) patients experienced infusion-related reactions. At an 18.5-month median follow-up, overall response rate was 43%. Median progression-free survival (PFS) and overall survival (OS) were 6.7 months and not reached, respectively; 12-month PFS and OS rates were 35% and 70%. Pharmacokinetic results (*n* = 22) were consistent with other studies. Safety, pharmacokinetics, and efficacy of daratumumab monotherapy were confirmed in Chinese patients with RRMM. This trial is registered on ClinicalTrials.gov (NCT02852837).

## Introduction

Based on a recent epidemiologic study, the estimated average age-adjusted prevalence of multiple myeloma (MM) in China was 5.68 per 100,000 population from 2012 through 2016, with an incidence of 1.15 per 100,000 person-years in 2016 [1]. Outcomes of patients with MM have improved with the introduction of novel treatment agents, including proteasome inhibitors (PIs) and immunomodulatory drugs (IMiDs) [2]. However, the majority of patients will relapse, and patients have limited treatment options after exposure to these classes of agents [3–5]. Patients who are heavily pretreated and/or refractory to both a PI and an IMiD have a poor prognosis [3–5], underscoring the need for efficacious, tolerable treatments with new mechanisms of action.

Daratumumab is a human IgGκ monoclonal antibody targeting CD38 with a direct on-tumor [6–9] and immunomodulatory [10–12] mechanism of action. In the global phase 3 CASTOR and POLLUX studies, daratumumab in combination with standard-of-care regimens consistently demonstrated significantly prolonged progression-free survival (PFS) and improved rates of deep responses, including minimal residual disease negativity, in patients with relapsed or refractory MM (RRMM) [13, 14], leading to the approval of intravenous (IV) daratumumab at 16 mg/kg in combination with standard-of-care regimens in many countries [15, 16]. The safety and efficacy of daratumumab 16 mg/kg IV monotherapy in patients with heavily pretreated RRMM were investigated in the global, first-in-human, phase 1/2 study GEN501 and global phase 2 SIRIUS studies [17, 18]. In GEN501, at a median follow-up of 10.2 months, daratumumab monotherapy demonstrated an overall response rate (ORR) of 36% and a median PFS of 5.6 months [17]. In SIRIUS, at a median follow-up of 9.3 months, ORR was 29% and median PFS was 3.7 months [18]. In a pooled, post hoc final analysis of GEN501 and SIRIUS, after a median follow-up of 36.6 months, patients with heavily pretreated RRMM receiving daratumumab monotherapy at 16 mg/kg achieved a median overall survival (OS) of 20.5 months (95% confidence interval [CI], 16.6–28.1) [19], consistent with an earlier pooled analysis of these studies [20]. Daratumumab monotherapy is associated with a manageable safety profile, with a low rate of treatment discontinuation due to adverse events (AEs) [19], and is also approved in many countries for the treatment of RRMM [15, 16].

Here, we present the results of the final analysis of MMY1003, the first study of daratumumab IV to be conducted specifically in Chinese patients. The MMY1003 study evaluated daratumumab IV monotherapy in Chinese patients who had received ≥ 2 prior lines of therapy, including a PI or an IMiD in parts 1 and 2, and in patients who had received both a PI and an IMiD, with documented disease progression on the last regimen, in part 3.

## Patients and methods

### Patients

This open-label, phase 1 study consisted of 3 parts: a dose-escalation part (part 1), a pharmacokinetic expansion part (part 2), and a safety expansion part (part 3). Inclusion criteria for parts 1 and 2 included ≥ 20 years of age; RRMM after receiving ≥ 2 prior lines of systemic therapy, which must have included a PI or an IMiD; an Eastern Cooperative Oncology Group performance status (ECOG PS) score ≤ 2; and a life expectancy of > 6 months. Exclusion criteria included prior exposure to daratumumab or other therapies targeting CD38; any antimyeloma treatment within 4 weeks or 5 pharmacokinetic half-lives of the treatment, whichever was longer, before the first administration of study drug; receipt of an allogeneic stem cell transplant; receipt of an autologous stem cell transplant within 12 weeks of administration of study drug; or a history of malignancy other than MM within 3 years of administration of study drug. Patients with primary refractory MM, absolute neutrophil count ≤ 1.0 × 10^9^ per L, hemoglobin concentration ≤ 7.5 g/dL, platelet count < 50 × 10^9^ per L (or < 75 × 10^9^ per L for patients in whom < 50% of bone marrow nucleated cells were plasma cells), alanine aminotransferase or aspartate aminotransferase level ≥ 2.5 × the upper limit of normal (ULN), total bilirubin level ≥ 2 × ULN, creatinine clearance ≤ 20 mL/min per 1.73 mm^2^, potassium level < 3.0 mEq per L, or corrected serum calcium > 14.0 mg/dL were excluded. Other exclusion criteria included meningeal involvement of MM; plasma cell leukemia, Waldenström’s macroglobulinemia, POEMS syndrome, or amyloidosis; clinically significant cardiac disease; chronic obstructive pulmonary disease with a forced expiratory volume in 1 s < 60% of predicted normal; and moderate or severe persistent asthma within the past 2 years or uncontrolled asthma of any classification.

Inclusion criteria for part 3 included ≥ 18 years of age, prior receipt of both a PI and an IMiD (each for ≥ 2 cycles or 2 months of treatment), and documented evidence of disease progression on or after the last regimen. Eligible patients had an ECOG PS score ≤ 2 and any of the following: absolute neutrophil count ≥ 1.0 × 10^9^ per L, hemoglobin concentration ≥ 7.5 g/dL, platelet count ≥ 50 × 10^9^ per L, creatinine clearance ≥ 20 mL/min per 1.73 mm^2^, aspartate aminotransferase ≤ 2.5 × ULN, alanine aminotransferase ≤ 2.5 × ULN, total bilirubin ≤ 2.0 × ULN, or corrected serum calcium ≤ 14.0 mg/dL or free ionized calcium ≤ 6.5 mg/dL. Patients were excluded if they had prior exposure to daratumumab or other therapies targeting CD38, any antimyeloma treatment within 2 weeks of Cycle 1 Day 1, nonsecretory MM, received an allogeneic stem cell transplant, or received an autologous stem cell transplant within 12 weeks of administration of study drug. Other exclusion criteria included a history of malignancy other than MM within 3 years of administration of study drug; meningeal involvement of MM; clinically significant cardiac disease; chronic obstructive pulmonary disease with a forced expiratory volume in 1 s < 50% of predicted normal; moderate or severe persistent asthma, a history of asthma within the past 2 years, or uncontrolled asthma of any classification; and plasma cell leukemia, Waldenström’s macroglobulinemia, POEMS syndrome, or amyloidosis.

### Study design and treatment

Part 1 had a modified 3 + 3 dose-escalation design with 3 treatment periods. In the first treatment period (weeks 1–3), 3 eligible patients received daratumumab at a starting dose of 8 mg/kg. This starting dose was chosen given that it is one-third of the maximum studied dose, which was shown to be safe and well tolerated in non-Chinese patients [17]. The dose-limiting toxicity (DLT) assessment period began with the first infusion of daratumumab and ended immediately before the initiation of the fourth infusion. If 1 of the 3 patients in a cohort experienced a DLT, then an additional 3 patients were to be treated at the same dose. The dose was to be escalated from 8 to 16 mg/kg only if none of the 3 patients in a cohort experienced a DLT. If none of the 3 patients in a cohort receiving 16 mg/kg experienced a DLT, then part 1 of the study was to be concluded and part 2 was to be initiated. The study was to be suspended if > 1 of the 3 patients in a cohort experienced a DLT or if ≥ 1 patient in the third cohort of patents treated at each dose level experienced a DLT. Patients received daratumumab as a single IV infusion on Cycle 1 Day 1, followed by a washout period of 3 weeks and safety monitoring. In the second treatment period (cycles 2–3 [weeks 4–9]), patients received 6 weekly doses of daratumumab IV in the third treatment period (cycles 4 + [weeks 10 +]), patients received daratumumab IV every 2 weeks for 8 infusions during cycles 4 through 7 (weeks 10–25), followed by once every 4 weeks from cycle 8 onward (weeks 26 +) until disease progression, intolerability, or other reasons for treatment discontinuation. Patients were hospitalized from the day prior to the first infusion up to 8 days after the first infusion for safety monitoring and pharmacokinetic sampling. Patients also had 1 day of hospitalization for the second, third, fourth, and seventh infusion of daratumumab for clinical observation and to facilitate pharmacokinetic sampling in the weekly dosing period.

During part 2, additional patients were enrolled until the total sample size in parts 1 and 2 was approximately 20 patients. Patients in part 2 were treated with daratumumab 16 mg/kg IV on the same treatment schedule used in part 1. In part 3, screening began after the last patient in part 2 received the first dose of daratumumab. Patients in part 3 were treated with daratumumab 16 mg/kg IV every week for 8 weeks (cycles 1–2), followed by every 2 weeks for an additional 16 weeks (cycles 3–6), and then every 4 weeks thereafter (cycles 7 +) until disease progression, intolerability, or other reasons for treatment discontinuation.

The study protocol and amendments were reviewed and approved by affiliated independent ethics committees and institutional review boards. The study was conducted in accordance with the ethical principles of the Declaration of Helsinki, the International Conference on Harmonisation Good Clinical Practice guidelines, and applicable regulatory requirements. All patients provided written informed consent to participate in this study.

### Endpoints and statistical analyses

Primary endpoints included safety (DLTs; part 1), pharmacokinetics (parts 1 and 2), and AEs. Secondary endpoints included ORR, time to response, duration of response, PFS, and OS.

The safety profile of daratumumab was evaluated by the incidence of AEs, deaths, laboratory results, vital signs, physical examination findings, electrocardiography results, and ECOG PS scores. The severity of AEs and the toxicity of laboratory parameters were assessed using the National Cancer Institute Common Terminology Criteria for Adverse Events (version 4.03) [21]. AEs were coded using the latest version of the *Medical Dictionary for Regulatory Activities* (MedDRA 22.1).

In parts 1 and 2, venous blood samples were collected on days 1, 4, 8, and 15 of cycle 1; days 1, 8, and 15 of cycle 2; days 1, 8, 15, and 18 of cycle 3; days 1 and 15 of cycles 4 through 7; day 1 of cycle 8 +; at the end of treatment; and at weeks 4 and 8 after the end of treatment to assess the serum concentration (pharmacokinetics) of daratumumab. The pharmacokinetic analysis population included all patients who had received ≥ 1 dose of daratumumab and had ≥ 1 post-infusion pharmacokinetic sample. All pharmacokinetic parameters (maximum observed serum concentration [C_max_], observed concentration just prior to start of a dosing interval [C_trough_], area under the concentration–time curve [AUC], total systemic clearance of drug after IV administration [CL], elimination half-life [t_1/2_], and volume of distribution of drug after IV administration [V]) were estimated using the actual sampling times by noncompartmental analysis methods. Baseline body weight was used for normalization. Descriptive statistics were used to summarize daratumumab serum concentrations at each sampling time point and pharmacokinetic parameters of daratumumab.

Efficacy analyses (ORR, time to response, duration of response, PFS, and OS) were performed based on the evaluation of response and disease progression using a validated computerized algorithm following the modified International Myeloma Working Group consensus recommendations for MM treatment response criteria [22, 23]. Efficacy analyses were also conducted in a prespecified subgroup of patients whose prior therapy included both a PI and an IMiD and who had demonstrated disease progression on their last therapy.

### Role of the funding source

Janssen Research & Development, LLC funded this study. The investigators and sponsor devised the study design and analyses. The investigators and their research teams collected the study data. The sponsor performed the final data analysis and confirmation of data accuracy. The investigators were not restricted by confidentiality agreements and had full access to the study data, participated in the development of this manuscript, and made the final decision to submit the manuscript for publication. Medical writing and editorial assistance were funded by Janssen Global Services, LLC.

### Data statement

The data sharing policy of Janssen Pharmaceutical Companies of Johnson & Johnson is available at https://www.janssen.com/clinical-trials/transparency. As noted on this site, requests for access to study data can be submitted through the Yale Open Data Access (YODA) Project site at http://yoda.yale.edu.

## Results

### Patient disposition and treatment

In part 1 of the study, 3 patients were first enrolled to receive daratumumab 8 mg/kg. After the 8 mg/kg dose level was determined to be safe and tolerable, another 3 patients were enrolled to receive 16 mg/kg daratumumab; no DLTs were reported at either dose level. Overall, of the 50 treated patients, 3 patients received 8 mg/kg daratumumab in part 1 and 47 patients received 16 mg/kg daratumumab (19 patients in parts 1 and 2 and 28 patients in part 3). After the 8 mg/kg and 16 mg/kg doses were both determined to be safe (no DLTs observed), all 3 patients in the 8 mg/kg group had their doses escalated to 16 mg/kg. The results from the 3 patients in the 8 mg/kg group who crossed over to receive 16 mg/kg daratumumab were included in their original 8 mg/kg group for all analyses and were not included in the 16 mg/kg group.

Demographic and baseline disease characteristics of all treated patients (*n* = 50) are summarized in Table 1. In the 16 mg/kg group (*n* = 47), the median (range) age was 61 (35–80) years, and 87.2% of patients had a baseline ECOG PS score ≤ 1. The median (range) time since the initial diagnosis of MM was 3.5 (0.5–10.1) years. High-risk cytogenetic abnormalities were observed in 3 (6.4%) patients. The median (range) number of prior MM therapies was 4.0 (2–11); 37 (78.7%) patients had previously received ≥ 3 lines of therapy, 46 (97.9%) patients had been previously treated with a PI, 47 (100%) patients had been previously treated with an IMiD, and 46 (97.9%) patients had been previously treated with both a PI and an IMiD. Thirty-seven (78.7%) patients were refractory to their last line of therapy, 21 (44.7%) patients were refractory to bortezomib, 30 (63.8%) patients were refractory to lenalidomide, and 15 (31.9%) patients were refractory to both a PI and an IMiD. Forty (85.1%) patients in the 16 mg/kg group were included in the subgroup of patients whose prior therapy included both a PI and an IMiD and who had demonstrated disease progression on their last therapy. For the 16 mg/kg group (*n* = 47), the median (range) duration of treatment was 5.8 (0–33.4) months; patients received a median (range) of 8.0 (1–38) daratumumab treatment cycles, with 24 (51.1%) patients receiving ≥ 8 cycles. The median (range) relative dose intensity of daratumumab was 99.2% (10.1–106.6%). At the clinical cutoff date (13 December 2019), all patients in the 16 mg/kg group had discontinued the study treatment. The most common reason for study treatment discontinuation was progressive disease (28 [59.6%] patients).

**Table 1:** Demographic and baseline disease characteristics

	Part 1 + part 2			Part 3	Total	
	8 mg/kg (*n* = 3)	16 mg/kg (*n* = 19)	Total (*n* = 22)	16 mg/kg (*n* = 28)	16 mg/kg (*n* = 47)	Total (*n* = 50)
Age, *n* (%)						
18– < 65 years	1 (33.3)	15 (78.9)	16 (72.7)	22 (78.6)	37 (78.7)	38 (76.0)
65–74 years	1 (33.3)	3 (15.8)	4 (18.2)	4 (14.3)	7 (14.9)	8 (16.0)
≥ 75 years	1 (33.3)	1 (5.3)	2 (9.1)	2 (7.1)	3 (6.4)	4 (8.0)
Median (range), years	66.0 (61–79)	58.0 (35–80)	60.5 (35–80)	61.0 (35–76)	61.0 (35–80)	61.0 (35–80)
Sex, *n* (%)						
Male	2 (66.7)	9 (47.4)	11 (50.0)	13 (46.4)	22 (46.8)	24 (48.0)
Baseline ECOG PS score, *n* (%)						
0	1 (33.3)	12 (63.2)	13 (59.1)	11 (39.3)	23 (48.9)	24 (48.0)
1	2 (66.7)	6 (31.6)	8 (36.4)	12 (42.9)	18 (38.3)	20 (40.0)
2	0	1 (5.3)	1 (4.5)	5 (17.9)	6 (12.8)	6 (12.0)
Median (range) time since initial diagnosis, years	3.6 (3.5–3.6)	3.5 (0.5–10.1)	3.5 (0.5–10.1)	3.5 (0.5–8.5)	3.5 (0.5–10.1)	3.5 (0.5–10.1)
Cytogenetic profile^a^, *n* (%)						
Standard risk	2 (66.7)	19 (100)	21 (95.5)	25 (89.3)	44 (93.6)	46 (92.0)
High risk	1 (33.3)	0	1 (4.5)	3 (10.7)	3 (6.4)	4 (8.0)
del17p	1 (33.3)	0	1 (4.5)	3 (10.7)	3 (6.4)	4 (8.0)
t(4;14)	0	0	0	1 (3.6)	1 (2.1)	1 (2.0)
t(14;16)	0	0	0	1 (3.6)	1 (2.1)	1 (2.0)
Number of prior lines of therapy						
< 3	0	5 (26.3)	5 (22.7)	5 (17.9)	10 (21.3)	10 (20.0)
≥ 3	3 (100)	14 (73.7)	17 (77.3)	23 (82.1)	37 (78.7)	40 (80.0)
Median (range)	3.0 (3–4)	4.0 (2–9)	3.5 (2–9)	4.5 (2–11)	4.0 (2–11)	4.0 (2–11)
Prior ASCT, *n* (%)	0	4 (21.1)	4 (18.2)	6 (21.4)	10 (21.3)	10 (20.0)
Prior PI, *n* (%)	3 (100)	18 (94.7)	21 (95.5)	28 (100)	46 (97.9)	49 (98.0)
Bortezomib	3 (100)	18 (94.7)	21 (95.5)	28 (100)	46 (97.9)	49 (98.0)
Prior IMiD, *n* (%)	3 (100)	19 (100)	22 (100)	28 (100)	47 (100)	50 (100)
Lenalidomide	3 (100)	17 (89.5)	20 (90.9)	24 (85.7)	41 (87.2)	44 (88.0)
Prior PI and IMiD, n (%)	3 (100)	18 (94.7)	21 (95.5)	28 (100)	46 (97.9)	49 (98.0)
Refractory to, n (%)						
Last prior line of therapy	3 (100)	13 (68.4)	16 (72.7)	24 (85.7)	37 (78.7)	40 (80.0)
Both PI and IMiD	0	7 (36.8)	7 (31.8)	8 (28.6)	15 (31.9)	15 (30.0)
Bortezomib	1 (33.3)	9 (47.4)	10 (45.5)	12 (42.9)	21 (44.7)	22 (44.0)
Lenalidomide	1 (33.3)	13 (68.4)	14 (63.6)	17 (60.7)	30 (63.8)	31 (62.0)

*ECOG PS*, Eastern Cooperative Oncology Group performance status; *ASCT*, autologous stem cell transplant; PI, proteasome inhibitor; *IMiD*, immunomodulatory drug

^a^Cytogenetic abnormalities were detected based on fluorescence in situ hybridization or chromosomal karyotype test results as reported by local laboratories

### Safety

No DLTs were observed in either dose level during the study. In the 16 mg/kg group (*n* = 47), all but 1 (97.9%) patient had ≥ 1 treatment-emergent adverse event (TEAE). Among the most common (occurring in ≥ 10% of patients) TEAEs were anemia (70.2%), leukopenia (59.6%), neutropenia (57.4%), lymphopenia (48.9%), upper respiratory tract infection (42.6%), and thrombocytopenia and hypokalemia (both 31.9%; see Table 2 for the full list of TEAEs occurring in ≥ 10% of patients). Thirty-six patients (76.6%) in the 16 mg/kg group experienced grade 3/4 TEAEs; the most common (occurring in ≥ 10% of patients) included anemia (29.8%), leukopenia (23.4%), neutropenia and thrombocytopenia (both 19.1%), lymphopenia (17.0%), hypokalemia (14.9%), and pneumonia (10.6%; Table 2).

**Table 2:** Most common (≥ 10%) TEAEs

	Part 1 + part 2						Part 3		Total			
	8 mg/kg (*n* = 3)		16 mg/kg (*n* = 19)		Total (*n* = 22)		16 mg/kg (*n* = 28)		16 mg/kg (*n* = 47)		Total (*n* = 50)	
	Any grade	Grade 3/4	Any grade	Grade 3/4	Any grade	Grade 3/4	Any grade	Grade 3/4	Any grade	Grade 3/4	Any grade	Grade 3/4
Patients with a TEAE, *n* (%)	3 (100)	2 (66.7)	18 (94.7)	13 (68.4)	21 (95.5)	15 (68.2)	28 (100)	23 (82.1)	46 (97.9)	36 (76.6)	49 (98.0)	38 (76.0)
Hematologic, *n* (%)												
Leukopenia	3 (100)	0	13 (68.4)	5 (26.3)	16 (72.7)	5 (22.7)	15 (53.6)	6 (21.4)	28 (59.6)	11 (23.4)	31 (62.0)	11 (22.0)
Anemia	2 (66.7)	0	12 (63.2)	4 (21.1)	14 (63.6)	4 (18.2)	21 (75.0)	10 (35.7)	33 (70.2)	14 (29.8)	35 (70.0)	14 (28.0)
Thrombocytopenia	2 (66.7)	0	5 (26.3)	4 (21.1)	7 (31.8)	4 (18.2)	10 (35.7)	5 (17.9)	15 (31.9)	9 (19.1)	17 (34.0)	9 (18.0)
Neutropenia	0	0	14 (73.7)	3 (15.8)	14 (63.6)	3 (13.6)	13 (46.4)	6 (21.4)	27 (57.4)	9 (19.1)	27 (54.0)	9 (18.0)
Lymphopenia	0	0	10 (52.6)	2 (10.5)	10 (45.5)	2 (9.1)	13 (46.4)	6 (21.4)	23 (48.9)	8 (17.0)	23 (46.0)	8 (16.0)
Nonhematologic, *n* (%)												
Hyperuricemia	3 (100)	1 (33.3)	2 (10.5)	0	5 (22.7)	1 (4.5)	4 (14.3)	0	6 (12.8)	0	9 (18.0)	1 (2.0)
Upper respiratory tract infection	2 (66.7)	1 (33.3)	9 (47.4)	2 (10.5)	11 (50.0)	3 (13.6)	11 (39.3)	2 (7.1)	20 (42.6)	4 (8.5)	22 (44.0)	5 (10.0)
Hypokalemia	2 (66.7)	1 (33.3)	8 (42.1)	3 (15.8)	10 (45.5)	4 (18.2)	7 (25.0)	4 (14.3)	15 (31.9)	7 (14.9)	17 (34.0)	8 (16.0)
Hypertension	1 (33.3)	0	3 (15.8)	2 (10.5)	4 (18.2)	2 (9.1)	2 (7.1)	0	5 (10.6)	2 (4.3)	6 (12.0)	2 (4.0)
Pneumonia	1 (33.3)	1 (33.3)	4 (21.1)	2 (10.5)	5 (22.7)	3 (13.6)	7 (25.0)	3 (10.7)	11 (23.4)	5 (10.6)	12 (24.0)	6 (12.0)
Increased AST	1 (33.3)	1 (33.3)	10 (52.6)	1 (5.3)	11 (50.0)	2 (9.1)	1 (3.6)	0	11 (23.4)	1 (2.1)	12 (24.0)	2 (4.0)
Increased ALT	1 (33.3)	0	8 (42.1)	1 (5.3)	9 (40.9)	1 (4.5)	1 (3.6)	0	9 (19.1)	1 (2.1)	10 (20.0)	1 (2.0)
Increased blood pressure	1 (33.3)	1 (33.3)	4 (21.1)	1 (5.3)	5 (22.7)	2 (9.1)	3 (10.7)	0	7 (14.9)	1 (2.1)	8 (16.0)	2 (4.0)
Pyrexia	1 (33.3)	0	4 (21.1)	0	5 (22.7)	0	7 (25.0)	0	11 (23.4)	0	12 (24.0)	0
Cough	1 (33.3)	0	3 (15.8)	0	4 (18.2)	0	5 (17.9)	0	8 (17.0)	0	9 (18.0)	0
Hypercalcemia	0	0	6 (31.6)	3 (15.8)	6 (27.3)	3 (13.6)	3 (10.7)	1 (3.6)	9 (19.1)	4 (8.5)	9 (18.0)	4 (8.0)
Hypoalbuminemia	0	0	5 (26.3)	2 (10.5)	5 (22.7)	2 (9.1)	3 (10.7)	0	8 (17.0)	2 (4.3)	8 (16.0)	2 (4.0)
Hypocalcemia	0	0	3 (15.8)	0	3 (13.6)	0	3 (10.7)	0	6 (12.8)	0	6 (12.0)	0
Back pain	0	0	4 (21.1)	2 (10.5)	4 (18.2)	2 (9.1)	2 (7.1)	0	6 (12.8)	2 (4.3)	6 (12.0)	2 (4.0)

*TEAE*, treatment-emergent adverse event; *AST*, aspartate aminotransferase; *ALT*, alanine aminotransferase

In the 16 mg/kg group (*n* = 47), 23 (48.9%) patients had serious TEAEs; the most frequently reported were pneumonia and thrombocytopenia (both occurring in 4 [8.5%] patients). One patient had a serious TEAE leading to treatment discontinuation (an infusion-related reaction [IRR] of dyspnea), and 1 patient had a TEAE leading to death (pneumonia); both occurred in part 3 of the study. Thirteen (27.7%) patients had treatment-emergent IRRs; most IRRs occurred during the first infusion. One patient had a grade 3 IRR of depressed level of consciousness, 1 patient had a grade 4 IRR of dyspnea, and 1 patient discontinued study treatment due to an IRR.

### Pharmacokinetics

**Fig. 1:**
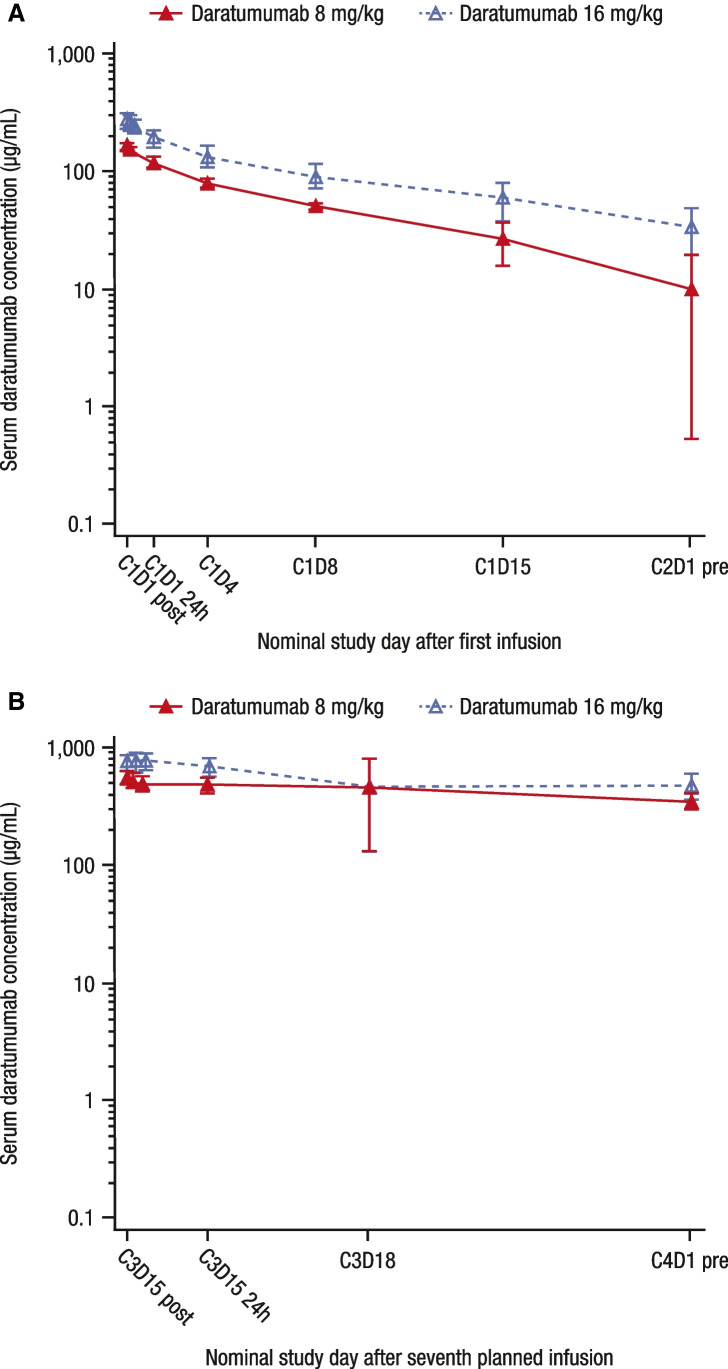
Mean (± SD) daratumumab serum concentration–time following the first infusion (**A**) and following the seventh planned infusion (**B**) in parts 1 and 2 of MMY1003. Post indicates sample taken immediately after infusion; 24 h indicates sample taken 24 h after infusion; pre indicates sample taken before infusion. SD, standard deviation; C, cycle; D, day; h, hour

At the first pharmacokinetic analysis cutoff date (9 November 2017), part 3 of the study remained ongoing; pharmacokinetics were evaluated in all patients in parts 1 and 2 (*n* = 22; Table 3). Following the first infusion, mean serum concentrations peaked around the end of infusion, and then declined in a biexponential manner and remained quantifiable through 21 days post-dose in most of the patients in both dose groups (Fig. 1a). Following the seventh planned infusion, the same biexponential declining trends were observed in both dose groups, with quantifiable concentrations at 7 days post-dose in all patients (Fig. 1b).

**Table 3:** Summary of pharmacokinetic parameters for parts 1 and 2

	8 mg/kg (*n* = 3)	16 mg/kg (*n* = 19)
Following first infusion		
C_max_ (μg/mL)		
Mean (SD)	171 (2.8)	276 (82.7)
AUC_0-7 day_ (h*μg/mL)		
Mean (SD)	14,832 (582)	24,939 (9,786)
AUC_last_ (h*μg/mL)		
Mean (SD)	24,820 (2,093)	44,446 (24,063)
t_1/2_ (hours)		
N	3	14
Mean (SD)	144 (71.6)	117 (70.6)
CL (mL/h/kg)		
N	3	14
Mean (SD)	0.296 (0.050)	0.635 (0.521)
V (mL/kg)		
N	3	14
Mean (SD)	58.3 (23.3)	71.3 (19.0)
Following seventh infusion		
C_max_ (μg/mL)		
N	3	16
Mean (SD)	592 (54.6)	803 (269)
C_trough_ (μg/mL)		
N	3	16
Mean (SD)	290 (44.1)	420 (194)
AUC_0-7 day_ (h*μg/mL)		
N	3	15
Mean (SD)	70,903 (9,048)	98,918 (40,786)
CL (mL/h/kg)		
N	3	15
Mean (SD)	0.114 (0.015)	0.205 (0.127)

*C*_*max*_, maximum observed serum concentration; *SD*, standard deviation; *AUC*, area under the concentration–time curve; *t*_*1/2*_, elimination half-life; *CL*, total systemic clearance of drug after intravenous administration; *V*, volume of distribution of drug after intravenous administration; *C*_*trough*_, trough serum concentration

Mean serum concentrations over time in the 16 mg/kg group were consistently higher than those in the 8 mg/kg group, despite the larger variation observed in the 16 mg/kg group (Fig. 1). In the 16 mg/kg group, accumulation of daratumumab continued throughout weekly dosing and decreased slightly as patients entered the every-2-week dosing period and the subsequent every-4-week dosing period.

Following the first infusion, C_max_ was approximately 1.6-fold higher in the 16 mg/kg group (276 μg/mL) than in the 8 mg/kg group (171 μg/mL), and mean AUC_last_ was approximately 1.8-fold higher in the 16 mg/kg group (44,446 h*µg/mL) than in the 8 mg/kg group (24,820 h*µg/mL; Table 3). Mean t_1/2_ values were comparable in the 2 dose groups (144 h in the 8 mg/kg group and 117 h in the 16 mg/kg group). The ranges of CL values were overlapping between the 2 dose groups, although mean CL values were higher with larger variation in the 16 mg/kg group (0.296 mL/h/kg for the 8 mg/kg group vs 0.635 mL/h/kg for the 16 mg/kg group). Mean V values were generally consistent across the dose levels (58.3 mL/kg in the 8 mg/kg group and 71.3 mL/kg in the 16 mg/kg group). Following the seventh planned infusion, mean C_max_ was approximately 1.4-fold higher in the 16 mg/kg group (803 μg/mL) than in the 8 mg/kg group (592 μg/mL), and mean AUC_0-7 day_ was approximately 1.4-fold higher in the 16 mg/kg group (98,918 h*µg/mL) than in the 8 mg/kg group (70,903 h*µg/mL; Table 3). Mean CL values were comparable in the 2 dose groups (0.114 mL/h/kg in the 8 mg/kg group and 0.205 mL/h/kg in the 16 mg/kg group). Mean C_max_ increased from Cycle 1 Day 1 to Cycle 3 Day 15 with weekly repeated dosing. In comparison with mean C_max_ following the first infusion, those following the seventh planned infusion were 3.5-fold and 2.9-fold higher in the 8 mg/kg group and the 16 mg/kg group, respectively. Mean CL values decreased following the seventh planned infusion, as compared to those following the first infusion.

### Efficacy

**Fig. 2:**
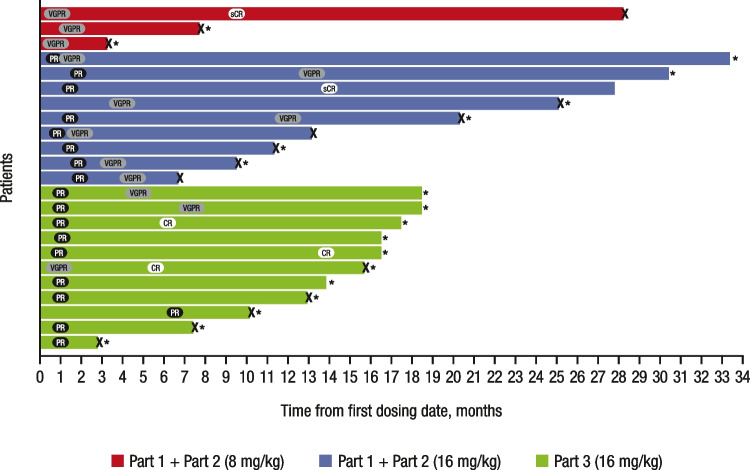
Swim-lane plot of patients who achieved ≥ PR in parts 1^a^, 2^b^, and 3^c^ in MMY1003. Patient disposition by hematologic response is shown for patients in part 1 plus part 2 who received daratumumab 8 mg/kg (red bars), patients in part 1 plus part 2 who received daratumumab 16 mg/kg (blue bars), and patients in part 3 who received daratumumab 16 mg/kg (gray bars). X indicates disease progression. The asterisk indicates patients whose prior therapy included both a proteasome inhibitor and an immunomodulatory drug and who had demonstrated disease progression on their last therapy. VGPR, very good partial response; sCR, stringent complete response; PR, partial response; CR, complete response. ^a^Patients who achieved ≥ PR in parts 1 + 2 (8 mg/kg dose group): *n* = 3 out of 3 total patients. ^b^Patients who achieved ≥ PR in parts 1 + 2 (16 mg/kg dose group): *n* = 9 out of 19 total patients. ^c^Patients who achieved ≥ PR in part 3 (16 mg/kg dose group): *n* = 11 out of 28 total patients

For the 16 mg/kg group (*n* = 47), the ORR was 42.6%, with 1 (2.1%) patient achieving stringent complete response (sCR), 3 (6.4%) achieving complete response (CR), 9 (19.1%) achieving very good partial response (VGPR), and 7 (14.9%) achieving partial response (PR; Fig. 2; Table 4). Responses continued to deepen over time. Notably, 1 patient in the 8 mg/kg group with an initial VGPR went on to achieve an sCR after crossing over to receive daratumumab 16 mg/kg. In the 16 mg/kg group, among the 18 patients with an initial PR, 8 patients went on to achieve a VGPR, 2 went on to achieve a CR, and 1 went on to achieve an sCR. Additionally, 1 patient with an initial response of VGPR went on to achieve a CR (Fig. 2). For the 16 mg/kg group, the median (range) time to first response was 1.0 (0.7–6.5) months. The median (range) time to best response was 4.2 (0.9–14.0) months. At a median follow-up of 18.5 months, the median duration of response was 18.9 months (95% CI, 7.62–not estimable [NE]).

**Table 4:** Overall best responses

	Part 1 + part 2		Part 3	Total
	8 mg/kg (*n* = 3)	16 mg/kg (*n* = 19)	16 mg/kg (*n* = 28)	16 mg/kg (*n* = 47)
Overall response rate, *n* (%)	3 (100)	9 (47.4)	11 (39.3)	20 (42.6)
Complete response or better	1 (33.3)	1 (5.3)	3 (10.7)	4 (8.5)
Stringent complete response	1 (33.3)	1 (5.3)	0	1 (2.1)
Complete response	0	0	3 (10.7)	3 (6.4)
Very good partial response or better	3 (100)	8 (42.1)	5 (17.9)	13 (27.7)
Very good partial response	2 (66.7)	7 (36.8)	2 (7.1)	9 (19.1)
Partial response	0	1 (5.3)	6 (21.4)	7 (14.9)
Minimal response, *n* (%)	0	2 (10.5)	2 (7.1)	4 (8.5)
Stable disease, *n* (%)	0	5 (26.3)	10 (35.7)	15 (31.9)
Progressive disease, *n* (%)	0	1 (5.3)	4 (14.3)	5 (10.6)
Not evaluable, *n* (%)	0	2 (10.5)	1 (3.6)	3 (6.4)

For the 16 mg/kg group (*n* = 47), as of the clinical cutoff date, 33 (70.2%) patients had progressive disease or died. The median PFS was 6.7 months (95% CI, 3.71–11.33), with an estimated 12-month PFS rate of 35.3% (95% CI, 21.5–49.4%; Table 5). A total of 19 (40.4%) deaths were observed. The median OS was not reached, with an estimated 12-month OS rate of 69.8% (95% CI, 54.4–80.9%; Table 5).

**Table 5:** PFS and OS

	Part 1 + part 2		Part 3	Total
	8 mg/kg (*n* = 3)	16 mg/kg (*n* = 19)	16 mg/kg (*n* = 28)	16 mg/kg (*n* = 47)
PFS				
Number of events (%)	3 (100)	14 (73.7)	19 (67.9)	33 (70.2)
Median, months (95% CI)	7.7 (3.19–28.25)	6.7 (5.59–20.27)	4.3 (1.87–12.91)	6.7 (3.71–11.33)
6-month PFS rate, % (95% CI)	-	71.3 (44.1–87.0)	42.7 (23.8–60.4)	54.1 (38.3–67.5)
12-month PFS rate, % (95% CI)	-	35.7 (14.7–57.5)	35.0 (17.7–52.9)	35.3 (21.5–49.4)
OS				
Number of events (%)	3 (100)	9 (47.4)	10 (35.7)	19 (40.4)
Median, months (95% CI)	25.1 (15.38–33.81)	NR (12.06–NE)	NR (7.43–NE)	NR (16.56–NE)
6-month OS rate, % (95% CI)	-	89.5 (64.1–97.3)	71.4 (50.9–84.6)	78.7 (64.1–87.9)
12-month OS rate, % (95% CI)	-	78.9 (53.2–91.5)	63.9 (43.3–78.7)	69.8 (54.4–80.9)

*PFS*, progression-free survival; *OS*, overall survival; *CI*, confidence interval; *NR*, not reached; *NE*, not estimable

Among patients in the 16 mg/kg subgroup whose prior therapy included both a PI and an IMiD and who had demonstrated disease progression on their last therapy (*n* = 40), the ORR was 42.5%, with 3 (7.5%) patients achieving CR, 7 (17.5%) achieving VGPR, and 7 (17.5%) achieving PR. The median (range) time to first response was 1.0 (0.7–6.5) months. The median (range) time to best response was 3.9 (0.9–13.8) months. The median duration of response was 18.9 months (95% CI, 7.62–NE). As of the clinical cutoff date, 28 (70.0%) patients had progressive disease or died. The median PFS was 6.0 months (95% CI, 2.76–12.91), with an estimated 12-month PFS rate of 35.5% (95% CI, 20.7–50.7%). As of the clinical cutoff date, 16 (40.0%) deaths were observed. The median OS was 26.5 months (95% CI, 20.60–NE), with an estimated 12-month OS rate of 67.1% (95% CI, 50.2–79.4%).

## Discussion

In MMY1003, at a median follow-up of 18.5 months, daratumumab IV monotherapy was well tolerated and showed encouraging clinical activity, with an ORR of 42.6%, in 47 Chinese patients with heavily pretreated RRMM. Responses to daratumumab 16 mg/kg monotherapy were rapid (median time to first response of 1.0 month), deep (≥ VGPR rate of 27.7%), and durable (median duration of response of 18.9 months) and continued to deepen over time. Efficacy results with daratumumab 16 mg/kg in the subgroup of patients whose prior therapy included both a PI and an IMiD and who had demonstrated disease progression on their last therapy were consistent with those observed in the overall population.

Daratumumab IV was initially approved as monotherapy in patients with heavily pretreated RRMM based on the favorable results of the global phase 1/2 GEN501 and phase 2 SIRIUS studies [17, 18]. While daratumumab-based combination regimens have also been approved in many countries based on the positive results observed in the phase 3 CASTOR and POLLUX studies [13, 14], many patients do not have access to these regimens, and single-agent therapy is often the sole option for RRMM patients who are refractory to agents from multiple drug classes. Given the positive results of global studies of daratumumab IV monotherapy in patients with heavily pretreated RRMM [17, 18], the MMY1003 study was conducted to evaluate daratumumab IV monotherapy specifically in Chinese patients. The results from parts 1 and 2 of this study supported the 2019 approval of daratumumab 16 mg/kg monotherapy in China for the treatment of patients with RRMM whose prior therapy included a both a PI and an IMiD and who have demonstrated disease progression on their last therapy.

The daratumumab IV doses (8 mg/kg and 16 mg/kg) used in MMY1003 were selected based on the results of the GEN501 study, which was the first-in-human study of daratumumab [17]. Results from MMY1003 demonstrate a favorable tolerability and safety profile of daratumumab monotherapy in Chinese patients with RRMM. No DLTs were observed at either dose level. Although the rates of anemia (70.2%), neutropenia (57.4%), and thrombocytopenia (31.9%) in the 16 mg/kg group were higher than those observed in a pooled analysis of GEN501 and SIRIUS (28.4%, 20.9%, and 21.6%, respectively), the safety profile demonstrated in this study was generally consistent with the known safety profile of daratumumab monotherapy [20]. A TEAE leading to treatment discontinuation and a TEAE leading to death were each observed in only 1 patient, with both occurring in part 3 of the study. IRRs were reported in 27.7% of patients in the 16 mg/kg group, with low rates of grade 3 and grade 4 IRRs and study treatment discontinuation due to IRRs.

The pharmacokinetic results from MMY1003 were consistent with those observed in other daratumumab studies [17, 24, 25]. Daratumumab exposure increased with increasing dose. Daratumumab elimination showed decreasing clearance following multiple doses in both dose groups. In the 16 mg/kg group, accumulation of daratumumab continued throughout weekly dosing and decreased slightly as patients entered the every-2-week dosing period and the subsequent every-4-week dosing period. The decreased clearance following the seventh infusion, possibly due to target-mediated drug disposition, is in line with previous reports of daratumumab pharmacokinetics [24]. Additionally, higher linear clearance and lower predicted C_trough_ of daratumumab at Cycle 3 Day 1 have been noted for RRMM patients with IgG versus non-IgG disease, likely due to competition for neonatal Fc receptor (FcRn) binding and protection from elimination between IgG M-protein secreted by myeloma cells and daratumumab; however, overall response rates with daratumumab were similar between IgG and non-IgG patients [26]. This dynamic has implications not only for daratumumab pharmacokinetics, but also for infection prophylaxis with intravenous immunoglobulin [27].

Results from this study demonstrated robust clinical efficacy of daratumumab IV monotherapy in Chinese RRMM patients who had failed ≥ 2 prior lines of therapy, as well as in the subgroup of patients whose prior therapy included both a PI and an IMiD and who had demonstrated disease progression on their last therapy, for whom there were limited effective or no approved therapies available in China. The overall efficacy data of daratumumab was consistent with that observed in global studies [20]. In a pooled analysis of the GEN501 and SIRIUS studies, the ORR was 31.1%, the ≥ VGPR rate was 13.5%, and the median time to and duration of response were 0.95 months and 7.6 months, respectively. The median PFS was 4.0 months, and the median OS was 20.1 months [20].

In summary, results from this phase 1 study in Chinese patients are consistent with those observed in global studies of daratumumab IV monotherapy in patients with heavily pretreated RRMM [19], with no new safety concerns observed. These results demonstrate the favorable benefit/risk profile in Chinese patients with heavily pretreated RRMM, including the subgroup of Chinese patients whose prior therapy included both a PI and an IMiD and who had demonstrated disease progression on their last therapy.

## Conclusion

Daratumumab IV was well tolerated in heavily pretreated Chinese patients at both the 8 mg/kg and 16 mg/kg dose levels; no new safety concerns were identified. The pharmacokinetic characteristics of daratumumab observed in Chinese patients were consistent with previous observations. The administration of daratumumab monotherapy demonstrated favorable efficacy, with an ORR of 42.6% in the 16 mg/kg group. Together, these data demonstrate a favorable benefit/risk profile of daratumumab monotherapy for the treatment of Chinese patients with RRMM who had failed ≥ 2 prior lines of therapy, as well as of Chinese patients whose prior therapy included both a PI and an IMiD and who had demonstrated disease progression on their last therapy.

## Data Availability

The data sharing policy of Janssen Pharmaceutical Companies of Johnson & Johnson is available at https://www.janssen.com/clinical-trials/transparency. As noted on this site, requests for access to study data can be submitted through the Yale Open Data Access (YODA) Project site at http://yoda.yale.edu.
